# Theoretical and Experimental Study on Hot-Embossing of Glass-Microprism Array without Online Cooling Process

**DOI:** 10.3390/mi11110984

**Published:** 2020-10-31

**Authors:** Manfeng Hu, Jin Xie, Wei Li, Yuanhang Niu

**Affiliations:** 1 School of Mechatronic Engineering and Automation, Foshan University, Foshan 528225, China; manfenghu@fosu.edu.cn (M.H.); liweigdut2007@163.com (W.L.); 2 School of Mechanical and Automotive Engineering, South China University of Technology, Guangzhou 510640, China; neilusmc1129@gmail.com

**Keywords:** hot-embossing, microprism array, optical glass, numerical simulation

## Abstract

Optical glass-microprism arrays are generally embossed at high temperatures, so an online cooling process is needed to remove thermal stress, but this make the cycle long and its equipment expensive. Therefore, the hot-embossing of a glass-microprism array at a low strain rate with reasonable embossing parameters was studied, aiming at reducing thermal stress and realizing its rapid microforming without online cooling process. First, the flow-field, strain-rate, and deformation behavior of glass microforming were simulated. Then, the low-cost microforming control device was designed, and the silicon carbide (SiC) die-core microgroove array was microground by the grinding-wheel microtip. Lastly, the effect of the process parameters on forming rate was studied. Results showed that the appropriate embossing parameters led to a low strain rate; then, the trapezoidal glass-microprism array could be formed without an online cooling process. The standard deviation of the theoretical and experimental forming rates was only 7%, and forming rate increased with increasing embossing temperature, embossing force, and holding duration, but cracks and adhesion occurred at a high embossing temperature and embossing force. The highest experimental forming rate reached 66.56% with embossing temperature of 630 °C, embossing force of 0.335 N, and holding duration of 12 min.

## 1. Introduction

An optical glass-microlens array has excellent micro-optical properties, and it is widely used in microfluidic chips [1], imaging [2], optical communication [3], solar-cell substrates [4], and other fields. For example, the glass microlens can be compactly integrated with a charge-coupled-device (CCD) camera to form a Shack¬–Hartmann sensor for wavefront measurement [5]. By employing a glass-microlens array integrated with subwavelength structures, the power-conversion efficiency of solar cell can be improved by 8.5% [6]. Refractive microlens arrays fabricated on the glass slide can reduce beam divergence and increase transmission [7]. Moreover, it was demonstrated that the glass microlenses can work in strong acid and high-temperature conditions in which other materials lenses cannot be used [8].

Due to its high glass-transition temperature and brittle fracture, it is difficult to precisely process a glass microlens [9]. Glass microgrooves are fabricated by using ion etching [10], ultrafast laser pulses [11], and micromilling technology [12]. However, most are relatively expensive and time-consuming. Compared to these fabrication methods, hot embossing is a net-shape approach for fabricating fine and high-precision microstructures on a glass substrate [13].

The dimensional accuracy of hot embossing depends on the precision of the die core [14], so research was carried out on core processing technology. Picosecond laser micromachining was used to fabricate a microcircular hole array on the steel die-core surface, but its machining accuracy was unknown [15]. The accuracy of the selective laser melting manufacturing of a metallic microstructured core was studied, but it had dimensional deviation [16]. Focused-ion-beam milling could produce high-surface-quality microstructures, but it had low processing efficiency [17]. Steel core micropits could also be prepared by chemical etching, but etching reagents easily pollute the environment [18]. Moreover, rectangular microchannels were milled on the aluminum tool surface, but it was easy to generate milling marks [19]. Microgrinding technology is an effective method for processing difficult-to-machine materials, but the effect of processing SiC die core has not been studied [20].

The hot embossing of glass microlens is challenging, so some related process studies were conducted [21,22]. The effect of the width of the die V-groove on the curvature and filled area of the molding glass microlens were investigated, but the influence of process parameters has not been studied [23]. The glass Fresnel lens was embossed with consideration of the process parameters, and indicated that the glass preform was easy to break under excessive molding force at low molding temperature [24]. Similarly, it was also revealed that the glass protrusion crack was generated at high imprinting pressure [25]. As a result, the crack affects the service life and performance of glass microstructures [26]. Therefore, it is necessary to optimize the process parameters.

Generally, the hot-embossing glass-microlens cycle is long due to its long cooling time to eliminate thermal stress [27]. A hexagonal-microlens array was fabricated via a precise thermal–mechanical embossing process, but the time of the glass-embossing process reached 138 min with cooling time of 88 min [28]. Refractive lens and diffractive gratings were developed, but the total time of hot embossing lasted about 250 min, including the cooling time of 120 min [29]. However, online cooling reduces efficiency, and water-cooling devices make the high-precision ultrathin glass-molding-process apparatus complex and expensive [30].

Theoretically, the hot-embossing process of a microprism array under different mold temperature, pressure, and holding-time levels was simulated by DEFORM software [31]. Finite-element analysis was also designed to evaluate the deformation behavior during hot-embossing processes [32,33]. Stress evolution in the cooling process was also simulated through finite-element simulation [34]. However, most of them are about polymer-microforming simulation, and there are few studies on the analysis of flow behavior, deformation, and strain rate during the hot embossing of glass microlenses [35].

In this paper, a hot-embossing method with low strain rate is proposed to realize the fabrication of a glass microlens array at low cost without an online cooling process. First, flow-field distribution, strain-rate change, and material-deformation process in the microforming process were simulated. Then, the microforming control device of the hot-embossing glass microlens was designed, and the grinding-wheel microtip was applied to fabricate a microgroove array on the surface of the SiC die core. Lastly, the effect of the process parameters on forming rate was studied.

## 2. Numerical Simulation

### 2.1. Simulation Model of Hot Embossing of Glass-Microprism Array

**Figure 1:**
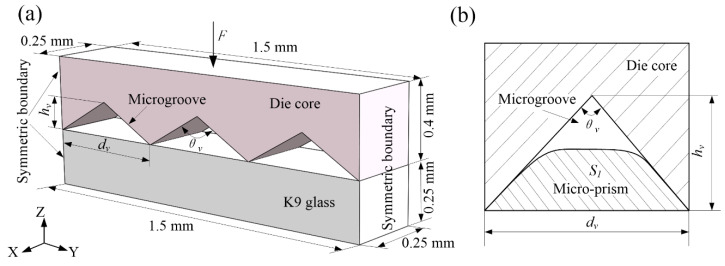
Simulation model of hot-embossing glass-microprism array: (**a**) physical-theory model; (**b**) formation of rate-calculation principle.

The simulation model of a hot-embossing glass-microprism array is shown in Figure 1. The die core was sized as 1.5 × 0.4 × 0.25 mm, and its surface was covered by microgrooves with angle *θ_v_* of 120° and height *h_v_* of 145 μm. The size of the glass substrate contacting with the microgroove was 1.5 × 0.25 × 0.25 mm. Both sides of the substrate were considered as symmetrical boundaries. The sparse solver was adopted, and the simulation time step length was set as 1 s. Moreover, preheating time and holding duration were given as 8 and 10 min, respectively. The sliding friction coefficient was regarded as 0.08. Simulation-environment temperature *T*, holding duration *t,* and embossing force *F* were set as 560–710 °C, 8–12 min, and 0.335–0.835 N, respectively. DEFORM software was adopted to carry out simulation research.

During the hot embossing of a glass-microprism array, the relationship between plastic flow stress σ, temperature *T*, strain ε, and strain rate $\dot{\varepsilon }$ is given in the following equation [36]:

(1) $$\sigma =f(\varepsilon ,\dot{\varepsilon },T)$$

Because there was no heat source inside the material, the temperature change in the hot-embossing process satisfied the simplified heat-balance equation:

(2) $$\rho c\frac{\partial T}{\partial t}=\frac{\partial }{\partial x}[k\frac{\partial T}{\partial x}],$$

where *c* is specific heat; and *ρ*, *T,* and *k* are density, temperature, and heat conductivity, respectively [37].

Because the cross-section of the die-core microgroove was triangular, the cross-sectional area of the microprism is given as *S*_1_; then, the forming rate during the forming process could be defined as

(3) $$\eta =2S_{1}/(h_{v}d_{v})$$

### 2.2. Material Properties

The substrate material was K9 optical glass with transformation temperature *T_g_* of 560 °C and the softening point *T_f_* of 714 °C. Transformation temperature *T_g_* means that the optical glass begins to be transformed from its solid state into a plastic state, and softening point *T_f_* indicates the temperature where the glass becomes obviously softened and deforms at its own weight [38]. The thermal conductivity and specific heat of the K9 optical glass were 5 W/(m·K) and 2100 J/(kg·K), respectively. The die-core material was SiC, which has high wear, temperature, and oxidation resistance. The thermal conductivity and specific heat of the optical glass were 77.5 W/(m·K) and 670 J/(kg·K), respectively. The material properties of the die core and the K9 optical glass substrate are listed in Table 1.

Table 2 shows flow stress σ for the K9 optical glass, which was adjusted to be consistent with our previous experiments, and set as the initial parameters in the simulation. This showed that flow stress *σ* increased with increasing strain rate $\dot{\varepsilon }$, and decreased with increasing temperature *T*.

## 3. Experiment and Measurement

### 3.1. Microgrinding of Core Microgrooves

**Figure 2:**
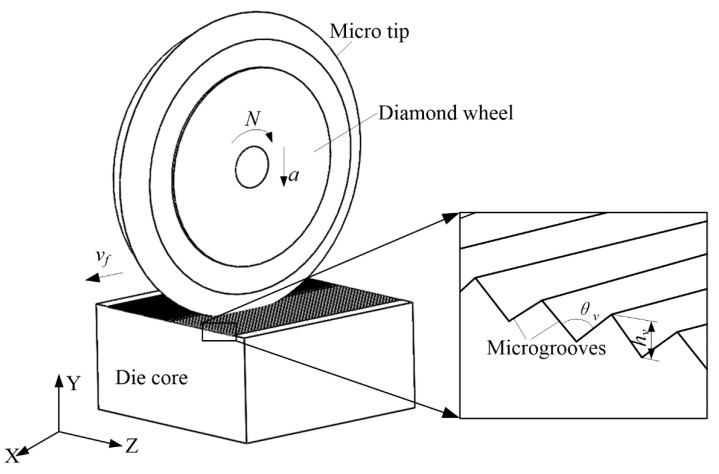
Scheme of microgrinding SiC die core.

The scheme of microgrinding SiC die core is shown in Figure 2. The SiC die core was sized as 30 × 30 × 10 mm, the resin-boned #3000 wheel was adopted to microgrind microgrooves on the die core, and microgroove angle *θ_v_* and depth *h_v_* were designed to be 120° and 145 μm, respectively. For form microgrinding, wheel speed *N*, speed rate *v_f_,* and cutting depth *a* were set as 2400 rpm, 500 mm/min, and 5 μm, respectively. After the cumulative cutting depth had reached 140 μm, finish microgrinding was carried out for 5 times, and feed rate *v_f_* and cutting depth *a* were changed to 100 mm/min and 1 μm, respectively.

### 3.2. Experiment Setup of Hot-Embossing Glass-Microprism Array

**Figure 3:**
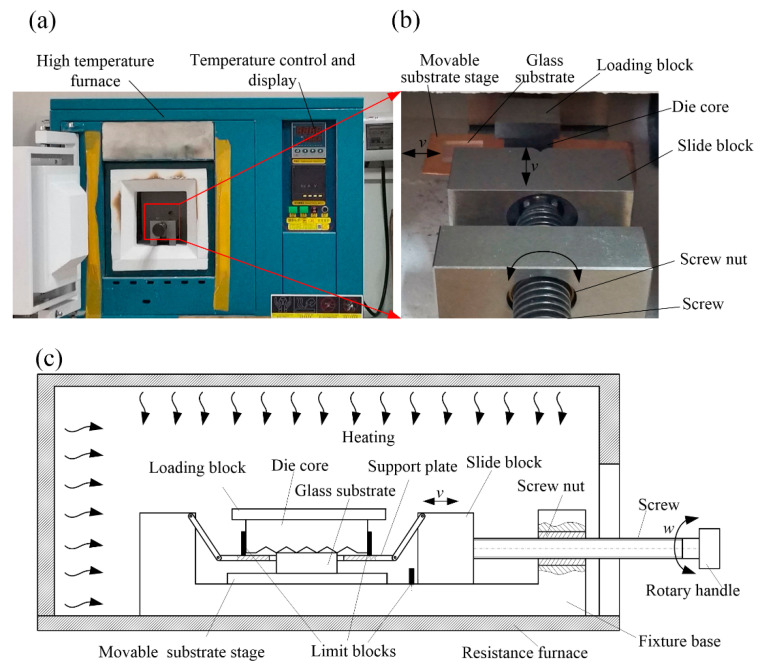
Experiment setup of hot-embossing glass-microprism array: (**a**) high-temperature furnace; (**b**) magnified view of experimental scene of hot-embossing glass-microprism array; (**c**) schematic diagram of principle of hot-embossing glass-microprism array.

The experiment setup of the hot-embossing glass-microprism array is shown in Figure 3. A high-temperature furnace was used to heat the die core and optical glass substrate in the hot-embossing experiment (see Figure 3a). The temperature sensor could detect temperature in the furnace in real time, and the maximal heating temperature of the furnace could reach 1200 °C. The console adopted an intelligent proportional-integral-derivative (PID) digital-display controller that could protect overload and short circuit.

Moreover, a small control device for hot-embossing glass-microprism array was designed and placed in the furnace. Figure 3b shows the magnified view of the experimental scene of the hot-embossing glass-microprism array, and Figure 3c shows the schematic diagram of the principle of the hot-embossing glass-microprism array. First, a K9 optical glass substrate with a size of 15 × 15 × 1 mm was placed on the movable substrate stage and moved to the work station. Then, the die core was placed on the support plate with a central hole; due to the limitation of the limit block around, the mold core could only move up and down on the support plate, not horizontally. The die core was moved over the substrate by rotating the rotary handle to control the relative movement between screw and screw nut. The substrate passed through the central hole of the support plate and closely contacted with the die core, as the height of the substrate was greater than the thickness of the support plate. Lastly, the hot-embossing force was adjusted by controlling the gravity of the loading block above the die core; the substrate was separated from the die core and sent out of the work station at the end of hot embossing.

Experimental parameters were set as embossing temperature *T* of 560–710 °C, holding duration *t* of 8–12 min, and embossing force *F* of 0.335–0.835 N.

Figure 3 shows that the experiment setup of the hot-embossing glass-microprism array was simple, cheap, and compact compared with that of the precision glass-molding machine (Guangdong HUST Industrial Technology Research Institute), which involves an numerical control - system, heating, bending, and annealing and cooling stages, a chamber, an entrance and exit mechanism, and ancillary facilities such as water-cooling and nitrogen-transport devices [30].

### 3.3. Processing of Hot-Embossing Glass-Microprism Array

**Figure 4:**
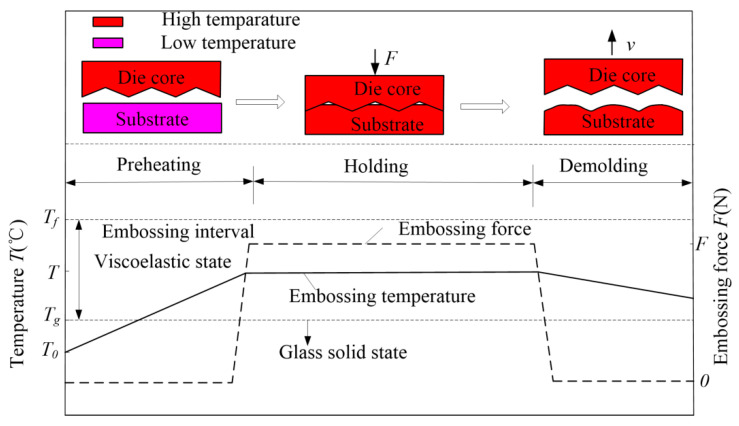
History of hot-embossing glass-microprism array.

The history of the hot-embossing glass-microprism array is shown in Figure 4. The whole process included preheating, holding, and demolding stages. The initial temperature of the substrate was at room temperature *T*_0_ = 20 °C, and it was preheated for about 5–10 min to achieve set embossing temperature *T*, which was greater than *T_g_* but less than *T_f_* because the glass was in viscoelastic state in this interval. Substrate and die core were separated after holding duration *t*, and the substrate was then cooled in air offline, while the die core was kept at a high temperature the entire time. Therefore, the die core did not undergo a cooling process, and it could avoid repeatedly switching on and off the power of the high-temperature furnace. This was different from the process of a hot-embossing glass microlens with an online cooling stage reported in other studies [39]. In this paper, embossing temperature *T*, holding duration *t,* and embossing force were set as 560–710 °C, 8–12 min, and 0.335–0.835 N, respectively.

## 4. Results and Discussion

### 4.1. Theoretical Analysis of Microforming Glass-Microprism Array Process

**Figure 5:**
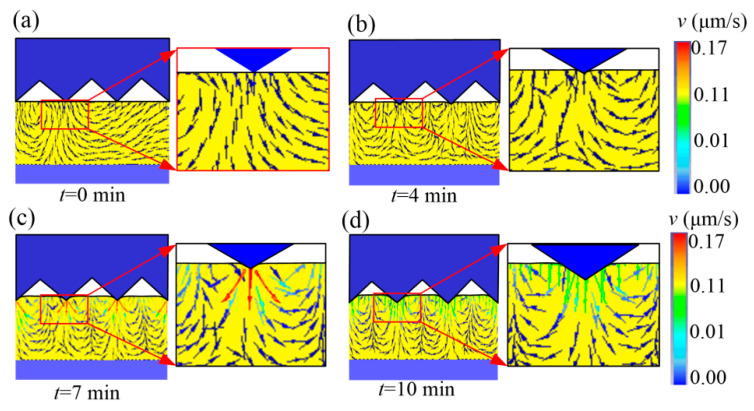
Flow-field distribution of microforming glass substrate. (**a**) flow-field distribution at *t* = 0 min; (**b**) flow-field distribution at *t* = 4 min; (**c**) flow-field distribution at *t* = 7 min; (**d**) flow-field distribution at *t* = 10 min.

The flow-field distribution of the microforming glass substrate is shown in Figure 5. In the simulation, hot-embossing parameters were set as holding duration *t* of 10 min, embossing temperature *T* of 630 °C, and embossing force *F* of 0.555 N. When the die-core tip entered the material, the molten glass material first flowed downward from the substrate (see Figure 5a), and then turned the flow direction toward the microgroove cavity, forming a vortex at *t* = 4 min (see Figure 5b). Flow rate in the forming process was very small, and the maximal velocity of 0.17 μm/s appeared at *t* = 7 min (see Figure 5c). Then, flow rate decreased to about 0.1 μm/s at *t* = 10 min (see Figure 5d). It can be deduced that the flow behavior of the molten polymer largely determines the shape of the glass microprism, and the polymer flowing into the microcavity of the die core is conducive to the formation of a glass-microprism array.

**Figure 6:**
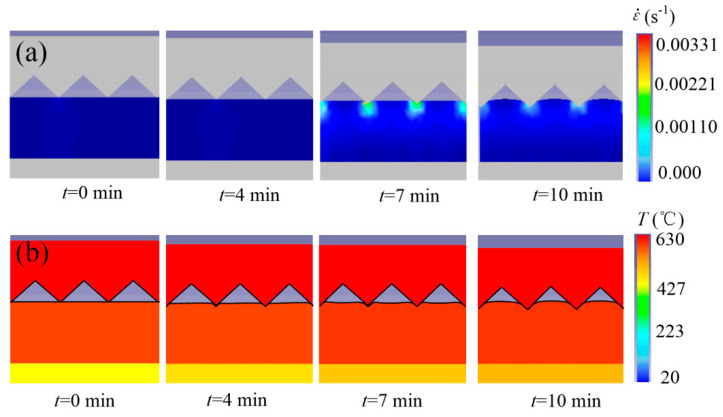
(**a**) Strain-rate and (**b**) deformation changes of microforming glass substrate.

Figure 6 shows the strain-rate and deformation change of the microforming glass substrate. The change of strain-rate is shown in Figure 6a. The strain-rate of the glass material was very small, the strain-rate of the polymer at the tip of the die-core microgroove tip was larger than that of other positions, and maximal strain-rate in the hot-embossing process was only 0.0011^−s^. It can be deduced from Equation (1) and Table 2 that a small strain-rate means small thermal stress, so a hot-embossing glass-microprism array may be realized through an offline air-cooling process instead of online cooling and annealing to remove thermal stress, which can save about 12–30 min of cooling and annealing time, compared with cooling and annealing the glass preform and mold from 560 °C to room temperature with a cooling rate of 0.25–0.75 °C/s [40].

The change of temperature field and deformation is shown in Figure 6b. After preheating, the temperature of the glass substrate reached about 600 °C, which was slightly lower than that of the die core (630 °C) because the thermal conductivity of the glass substrate was lower than that of the die core. As for the deformation of glass material, the substrate material was first squeezed into the cavity of the core microgroove, and a microprism array was then formed in the microgroove cavity. This process was similar to that of the microforming polymer microlens array reported in [41].

### 4.2. Microground Die Core

**Figure 7:**
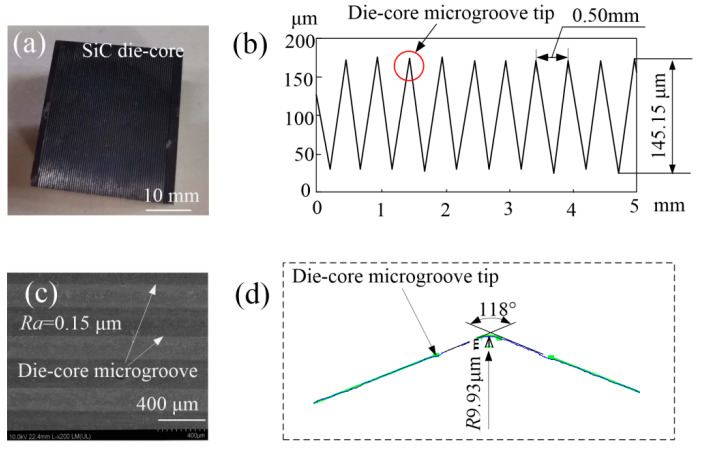
Microground die core: (**a**) photo of SiC die core; (**b**) surface profile; (**c**) SEM morphology of microgrooves on die-core surface; (**d**) angle *θ_v_* and radius *R* of die-core microgroove tip.

The microground die core is shown in Figure 7. The photo of the die core is shown in Figure 7a; it is shown that some continuous microgroove arrays were machined on the surface of the die core. Average total depth *h_v_* and width *d_v_* of the microgroove were 145.15 μm and 0.50 mm, respectively (see Figure 7b). The SEM morphology of the microgrooves on the die-core surface is shown in Figure 7c; the microground die-core microgroove surface was relatively smooth with a roughness of 0.15 μm. Angle *θ_v_* of the microgroove was 118°, compared with the designed angle of 120° and forming angle error of 1.7%. The radius of the microgroove tip was 9.93 μm, which was mainly caused by the wear of the grinding-wheel tip during the machining process (see Figure 7d).

### 4.3. Photograph and Profile of Glass-Microprism Array

**Figure 8:**
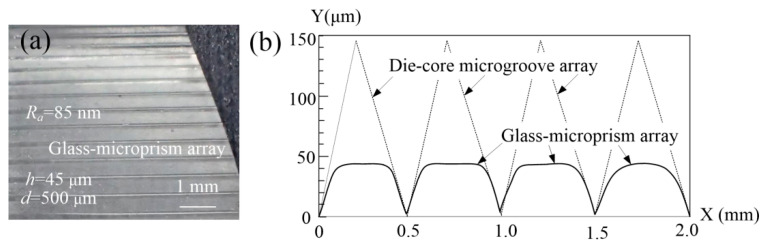
Photograph and profile of glass-microprism array: (**a**) photo of glass-microprism array; (**b**) cross-sectional profile of glass-microprism array (embossing temperature, 630 °C; holding duration, 10 min; embossing force, 0.555 N).

The photograph and profile of the glass-microprism array are shown in Figure 8. Processing parameters were set as embossing temperature of 630 °C, holding duration of 10 min, and embossing force of 0.555 N. The experiment showed that the glass-microprism array could be hot-embossed with a surface roughness of 85 nm; a photograph of the microformed glass-microprism array is shown in Figure 8a.

Moreover, the cross-sectional profile of the glass-microprism array is shown in Figure 8b; height *h* and width *d* of the microprism were 45 and 500 μm, respectively. According to Equation (3), forming rate *η* was calculated to be 50%. The shape of the microprism was approximately trapezoidal, which was consistent with simulation analysis (see Figure 6b). Therefore, this proves that the glass-microprism array could be molded without online cooling, and molding efficiency was significantly improved compared with the micro-hot-embossing time of 138 min in [28].

### 4.4. Theoretical and Experimental Forming Rates of Microprism Array

The theoretical and experimental forming rates of the glass-microprism array are shown in Figure 8. Theoretical values were in good agreement with the experimental values, and standard deviation and difference were controlled within 7% and 12%, respectively.

**Figure 9:**
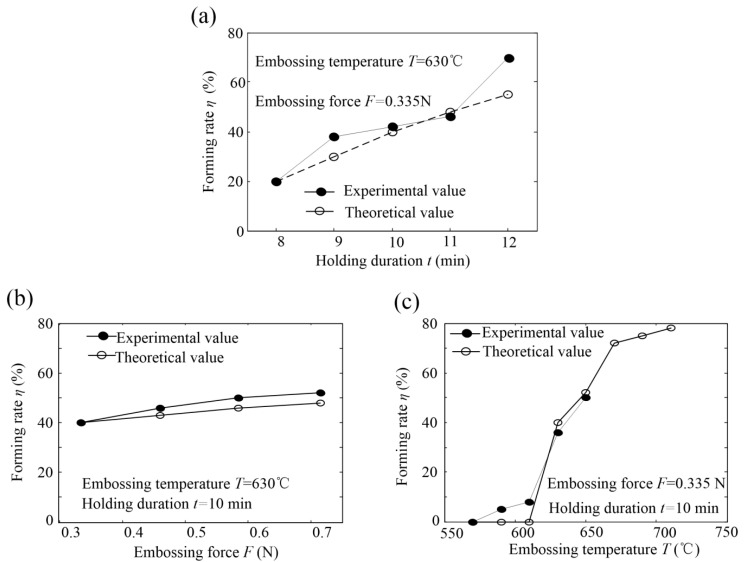
Theoretical and experimental forming rates of glass-microprism array: (**a**) forming rate *η* versus holding duration *t*; (**b**) forming rate *η* versus embossing force *F*; (**c**) forming rate *η* versus embossing temperature *T*.

When embossing temperature and force were 630 °C and 0.335 N, respectively, forming rate *η* increased with an increase in holding duration *t* (see Figure 9a). Forming rate was increased to 66.56% for holding duration *t* = 12 min, which was 3.5 times of the forming rate for holding duration *t* = 8 min. This is because an increase in holding time facilitated the flow of the molten-glass material into the microgrooves.

Figure 9b also shows that the greater the embossing force was, the greater the forming rate. This may be because the external force can accelerate the flow of molten glass and improve filling efficiency. Forming rate was about 50% for embossing force *F* = 0.715 N, which was about 1.25 times that for embossing force *F* = 0.355 N. However, compared with other hot-embossing parameters, embossing force had little effect on the microforming glass-microprism array.

Moreover, forming rate increased in “S” shape with the increase in embossing temperature; it first slowly increased, and then sharply increased when embossing temperature was higher than 610 °C (see Figure 9c). After the temperature had exceeded 650 °C, the theoretical result showed that the forming rate of the microprism array could reach 80%, while the K9 optical glass substrate cracked during the experiment. This may be because the higher the embossing temperature is, the higher the degree of melting and the higher the forming rate are. However, if the temperature were too high, the greater the thermal stress accumulated inside the substrate was, especially near the tip of the die-core microgroove, as shown in Figure 6a. This means that thermal-stress accumulation needs be precisely limited.

Therefore, it can be deduced from the theoretical and experimental results that embossing temperature had the greatest influence on forming rate, and embossing force had the least influence. Under a certain external force, the higher the embossing temperature is, the stronger the fluidity of the viscous-flow-state glass material, and higher forming rate, but too-high embossing temperature lead to thermal-stress accumulation and brittle substrate cracks.

### 4.5. Forming Defects during Hot-Embossing Glass-Microprism Array Process

**Figure 10:**
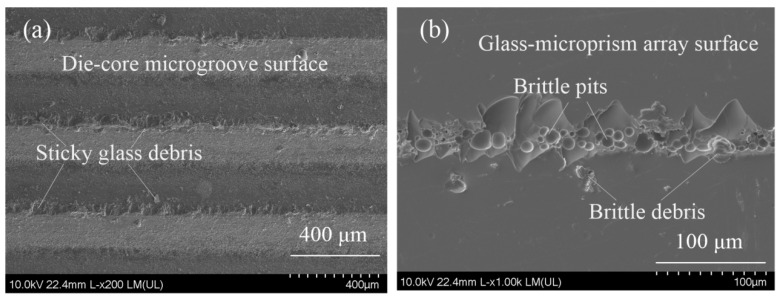
Forming defects during hot-embossing glass-microprism array process: (**a**) glass debris sticking on surface of die-core microgrooves; (**b**) brittle pits and debris on glass-microprism array surface (embossing temperature 700 °C, holding duration 10 min, embossing force 0.755 N).

Figure 10 shows the forming defects during the hot-embossing glass-microprism array process. When embossing temperature was close to the glass-softening point *T_f_* of 714 °C, the sticky glass debris stuck to the die-core microgroove tip (see Figure 10a). This may be due to the high adhesion of glass material at high temperatures. As shown in Figure 10b, brittle pits and debris appeared on the surface of the glass-microprism array, and the brittle pits were circular in the middle and irregular at the edge of the glass microstructure, which may have been caused by uneven thermal stress, as the reference reported that excessive forming stress and residual stress induce the growth of glass cracking [42]. Therefore, it is very important to control the appropriate embossing temperature and embossing force.

## 5. Conclusions

(1) Theoretical-simulation results showed that the vortexlike molten-glass material flowed into the core cavity to form a trapezoidal microprism array. Appropriate hot-embossing force and temperature led to small strain rate, which meant that thermal stress was low, and the online cooling process may not have been needed, reducing hot-embossing time.
(2) A low-cost microforming control device was designed, and the SiC die-core microgroove array was microground by the grinding-wheel microtip. Moreover, the trapezoidal microprism array was successfully embossed without online cooling processing, and the highest experimental forming rate reached 66.56% with a holding duration of 12 min.
(3) Theoretical and experimental values were in good agreement with standard deviation and difference of 7% and 12%, respectively. Theoretical and experimental results showed that the forming rate of the glass-microprism array increased with increasing holding duration, embossing force, and embossing temperature.
(4) When embossing temperature and embossing force were high, the hot-melt glass material was stuck to the die-core microgroove surface, and the optical glass substrate cracked, which may have been caused by uneven thermal stress.

## Figures and Tables

**Table 1:** Material properties of die core and K9 optical glass [38].

Material	Density (kg/m^3^)	Thermal Conductivity (W/(m·K))	Specific Heat (J/(kg·K))	Young’s Modulus (GPa)	Poisson’s Ratio
SiC die core	3.02 × 10^3^	77.5	670	192	0.142
K9 optical glass	2.41 × 10^3^	5	2100	79.2	0.211

**Table 2:** Flow stress σ for K9 optical glass.

$\dot{\varepsilon }/T$	503 °C	603 °C	703 °C	803 °C
0.0001	967	100	1.42 × 10^−5^	2.98 × 10^−7^
0.01	96,760	10,000	1.42 × 10^−3^	2.98 × 10^−5^
1	9,676,000	1,000,000	0.142	2.98 × 10^−3^
